# Giant Cell Tumor of the Frontal Bone: A Case Report and Literature Review

**DOI:** 10.7759/cureus.3353

**Published:** 2018-09-24

**Authors:** Shaheen Jadidi, Anthony D'Abarno, Jeanne F Barkley, Raed F Abusuwwa

**Affiliations:** 1 Physical Medicine and Rehabilitation, Northwestern University Feinberg School of Medicine, Chicago, USA; 2 Miscellaneous, Chicago College of Osteopathic Medicine, Schaumburg, USA; 3 Neurosurgery, Advocate Good Samaritan Hospital, Downers Grove, USA

**Keywords:** neurosurgery, giant cell tumour, calvarial tumors, oncology, osteoclastoma

## Abstract

Giant cell tumors are rare benign lesions that typically occur at the epiphyses of long bones in the extremities and present with pain or swelling. These lesions very seldom occur in the skull, where they preferentially affect the sphenoid and temporal bones that develop by endochondral ossification. We report a rare case of a giant cell tumor of the frontal bone and review the literature on these lesions. A 21-year-old woman presented with localized swelling and tenderness over the left frontal bone. Imaging revealed a lytic lesion involving the left frontal bone, which was managed via left frontal craniectomy with resection of the bone and epidural mass. Histopathology revealed a giant cell tumor of bone (GCTB). Most data on giant cell tumors in the skull consist of case reports, with many large series of giant cell tumors having no examples in the skull. This report contributes to the scarce literature on these tumors in the skull.

## Introduction

Giant cell tumor of bone (GCTB) represents approximately three to five percent of all primary bone tumors in the United States, yet it represents 20 percent of all primary bone tumors in China [1-4]. Traditionally, GCTB manifests in adults within the epiphyses of the long bones, and it is slightly more common in females [1,5-6]. The distal femur and proximal tibia are prototypically affected. The lungs are the most common site of metastases, which occur in about two to three percent of cases [7]. Malignant transformation of GCTB has also been reported [8-11]. There may be a hereditary component to the development of GCTB, especially of the skull and pelvis, in patients with Paget disease [12-13]. Recent studies have identified distinct genetic backgrounds between isolated GCTB versus GCTB associated with Paget disease, resulting in specific biochemical and histological characteristics of the tumor [14]. Less than one percent of the traditional GCTB cases are associated with multiple lesions [15]. Approximately 25 percent of GCTB associated with Paget disease occur as multiple lesions, and 75 percent of the cases associated with Paget disease affects the appendicular skeleton [14]. GCTB is characterized microscopically by abundant epithelioid to spindle-shaped mononuclear cells and evenly distributed large osteoclast giant cells, and histologic grading has little clinical value in predicting the tumor behavior [16]. However, recent evidence suggests there may be value in the genetic and histological characterization of GCTB to rule out the possible association with Paget disease, especially in cases that affect the skull or pelvis [14].

## Case presentation

Patient presentation

A 21-year-old female college student with a history of asthma presented to the neurosurgery office for consultation complaining of mass on the left side of her skull associated with increasing size over the past two days and intermittent headaches for the past two to three weeks. The left-sided headache included her upper jaw. She also reported a history of cellulitis and urinary tract infections, in addition to surgical removal of an impacted wisdom tooth in 2016. Family history was positive for diabetes mellitus (DM) type II in both her father and her grandfather and colon cancer and coronary artery disease in her other grandfather. She admitted to drinking alcohol one to two times per week but denied use of tobacco and drugs. At the time, she was taking Viorele birth control to regulate her menses. Review of systems was otherwise negative.

Clinical findings

Physical examination revealed a well-developed, well-nourished female in no acute distress. She was awake, alert and oriented to person, place and time with a Glasgow Coma Score (GCS) of 15. A soft left frontal lesion associated with tenderness to palpation, without erythema or drainage, was palpated slightly off midline. Her cranial nerves II-XII were intact. Strength in both upper and lower extremities was five out of five bilaterally. No pronator drift was noted. Sensation to light touch was intact bilaterally in V1-3, upper extremity, and lower extremity distributions. Her reflexes were symmetric. Her gait was within the normal limits. 

Imaging

**Figure 1:**
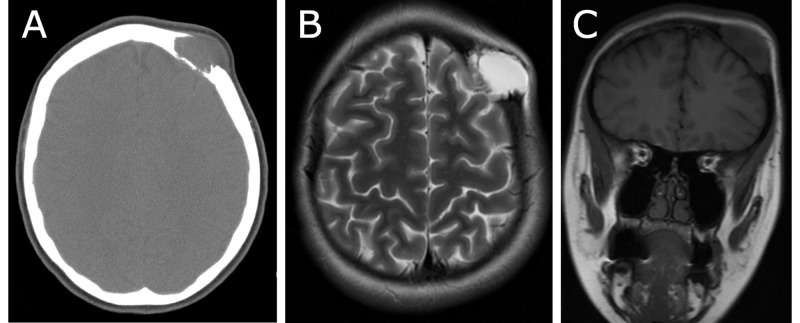
CT scan and MRI prior to surgery. A: Axial CT scan of the head without contrast, B: Axial T2-weighted MRI scan of the head, C: Coronal T1-weighted MRI scan of the head. CT: computed tomography; MRI: magnetic resonance imaging

CT of the head without contrast (Figure 1A) revealed an expansive soft tissue mass with beveled edges and dimensions measuring approximately 3.5 x 2.1 x 2.3 cm in the left frontal calvarium. Bony destructive changes of the inner and outer table of the left frontal calvarium were apparent. Extension of the mass into the dura was noted. The mass did not extend into the brain parenchyma. Magnetic resonance imaging (MRI) scans of the brain revealed a lytic bony lesion with dimensions measuring 2.4 x 2.9 x 2.9 cm, which was centrally T2 hyperintense (Figure 1B), peripherally enhancing, and T1 hypointense (Figure 1C). There appeared to be no hemodynamically significant effects of the mass on intracranial circulation. The mass did not appear to be hypervascular, and the bridging veins adjacent to the inner table of the calvarium were not hypertrophied or invaded by the mass. The adjacent dura of the left frontal lobe was thickened and enhancing, as well as the overlying subgaleal aponeurosis. MRI of the spine with and without contrast revealed a T2 hyperintense lesion measuring 0.6 cm x 0.8 cm in the sagittal plane at the anterior superior C7 vertebral body, a small hyperintensity at the superior T5 level, and a small hyperintensity at the S1 level, all of which were likely benign hemangiomas. X-ray bone survey of the whole body revealed no other suspicious lytic bone lesions.

Surgical management

**Figure 2:**
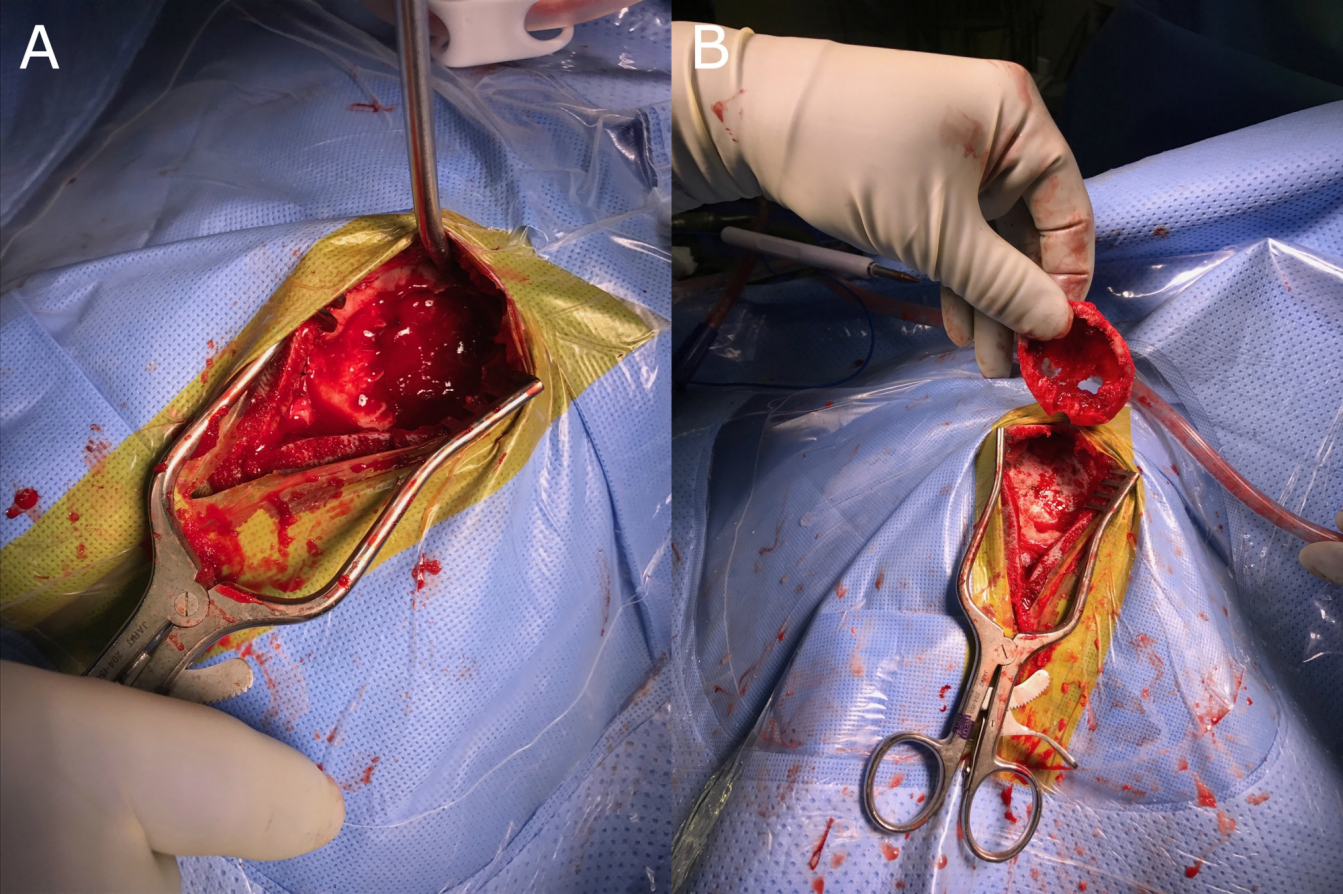
Intraoperative images. A: Image revealing curettage or scraping of the lesion which kept the excision of the tumor within the walls or the capsule of the tumor, B: A wide excision involving removal of any remaining tumor, reactive zone and some normal bone tissue. All specimens are sent to pathology to be examined for microscopic cells left behind in any of the margins. Results are reported as positive or negative margins. If margins are positive, cells have been left behind and additional excision may be necessary.

A left craniectomy for biopsy and resection of the calvarial lesion was performed (Figure 2), and the skull convexity was reconstructed with a titanium mesh. Three specimens were examined for pathological characterization. The patient was discharged home the following afternoon and scheduled for follow-up in two weeks for wound check.

Pathology report

**Figure 3:**
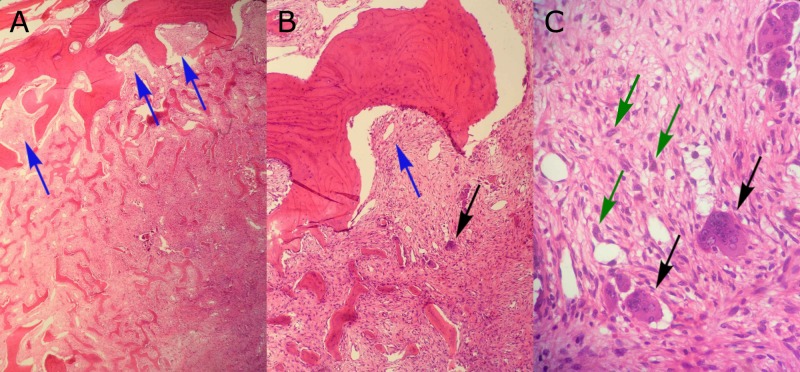
Histological results of pathology studies. The biopsy revealed extension of the tumor into bone (A, B; arrows). Numerous multinucleate, osteoclast-like giant cells (B, C; black arrows) were seen scattered throughout the mononuclear cells in the surrounding stroma. The mononuclear cells were noted to range in shape from spindle-like to rounded (C; green arrows) with the presence of large nuclei and prominent nucleoli.

The first specimen was received fresh and consisted of two pieces of pink fibromembranous tissue measuring 1.2 x 0.3 x 0.1 cm and 1.1 x 0.3 x 0.1 cm. The entire specimen was submitted for frozen section. The second specimen was received in formalin and consisted of a pink-purple tissue weighing 3.1 g and measuring 3.5 x 1.5 x 1.0 cm. The surface was partially covered with a semi-translucent membrane. The entire second specimen was submitted in four cassettes for histologic examination. The third specimen was fixed in 10% buffered formalin and consisted of a fragment of a bone measuring 5.0 x 4.0 cm excised to the depth of 0.9 cm. A depression measuring 3.7 x 2.5 cm with the depth of 0.8 cm was observed at the center of the bone. Representative sections of the third specimen were submitted in two cassettes for decalcification. The tumor was seen extending into bone (Figure 3A). All three specimens were diagnosed as GCTB, as they contained osteoclast-like multinucleated giant cells, round mononuclear cells, and spindle-shaped, fibroblast-like mononuclear cells (Figures 3B-3C).

## Discussion

Skull lesions include both benign (common) and malignant (rare) pathologies. Benign lesions include fibrous dysplasia, osteoid osteomas, or dermoid/epidermoid lesions and are generally painless and slow growing. Of the benign bony tumors, GCTB accounts for approximately 20%. Rapidly growing and painful lesions are at a higher risk of malignancy. In this case, the painful, rapidly growing mass was concerning for malignancy, and the differential diagnosis included a primary or metastatic neoplasm. Other differential diagnoses that were considered in this case were eosinophilic granuloma, epidermoid cyst, dermoid cyst, hemangioma, and infection.

GCTBs are very rarely skull tumors, with only one percent occurring in the skull. These tumors develop by endochondral ossification and are most commonly found in the sphenoid and temporal bones when found in the skull – both of which develop by endochondral ossification [17]. Our patient’s tumor is unique in that it arose from her left frontal bone that develops purely by intramembranous ossification.

GCTBs typically occur in the third and fourth decades of life [18], which is in contrast with our patient who was 21 years of age. Surgical resection is usually curative, assuming that full resection can be achieved. In this case, the size and location were favorable for a gross total resection. Radiation therapy is controversial in this entity and may lead to malignant degeneration in the future. In situations where cranial GCTBs cannot be completely excised, radiotherapy often becomes necessary to remove the remaining tumor. As adjuvant therapy, chemotherapy has been given in some cases with good results [19], but there is no well-defined accepted protocol for chemotherapy treatment in GCTB [20].

## Conclusions

Giant cell tumors are benign, locally expansive lesions with the potential to metastasize. This report contributes to the scarce literature on these tumors in the skull. These lesions generally present with pain and swelling. Radiographically, they appear as radiolucent lesions without sclerotic borders, which often appear in the sphenoid bone that is in contrast with our case in which the tumor manifested in the frontal bone. There is a higher female predominance and a slightly higher age at presentation in giant cell tumors of the skull compared to other sites. Because of their tendency to appear in the sphenoid bone, these lesions can present with cranial nerve deficits related to cavernous sinus involvement. There were no associated deficits in our patient's frontal bone tumor. Surgical excision is the treatment of choice and was successfully completed in our patient. Radiographic and histologic grading systems do not predict the clinical outcome, while the extent of surgical resection has been shown to be predictive of prognosis.

## References

[1] Larsson SE, Lorentzon R, Boquist L (1975). Giant-cell tumor of bone. A demographic, clinical, and histopathological study of all cases recorded in the Swedish Cancer Registry for the years 1958 through 1968. J Bone Joint Surg Am.

[2] Baena-Ocampo Ldel C, Ramirez-Perez E, Linares-Gonzalez LM (2009). Epidemiology of bone tumors in Mexico City: retrospective clinicopathologic study of 566 patients at a referral institution. Ann Diagn Pathol.

[3] Guo W, Xu W, Huvos AG, Healey JH, Feng C (1999). Comparative frequency of bone sarcomas among different racial groups. Chin Med J (Engl).

[4] Sung HW, Kuo DP, Shu WP, Chai YB, Liu CC, Li SM (1982). Giant-cell tumor of bone: analysis of two hundred and eight cases in Chinese patients. J Bone Joint Surg Am.

[5] Werner M (2006). Giant cell tumour of bone: morphological, biological and histogenetical aspects. Int Orthop.

[6] Viswanathan S, Jambhekar NA (2010). Metastatic giant cell tumor of bone: are there associated factors and best treatment modalities?. Clin Orthop Relat Res.

[7] Balke M, Schremper L, Gebert C (2008). Giant cell tumor of bone: treatment and outcome of 214 cases. J Cancer Res Clin Oncol.

[8] Domovitov SV, Healey JH (2010). Primary malignant giant-cell tumor of bone has high survival rate. Ann Surg Oncol.

[9] Grote HJ, Braun M, Kalinski T (2004). Spontaneous malignant transformation of conventional giant cell tumor. Skeletal Radiol.

[10] Brien EW, Mirra JM, Kessler S, Suen M, Ho JKS, Yang WT (1997). Benign giant cell tumor of bone with osteosarcomatous transformation ('dedifferentiated' primary malignant GCT): report of two cases. Skeletal Radiol.

[11] Anract P, De Pinieux G, Cottias P, Pouillart P, Forest M, Tomeno B (1998). Malignant giant-cell tumours of bone. Clinico-pathological types and prognosis: a review of 29 cases. Int Orthop.

[12] Rendina D, Mossetti G, Soscia E (2004). Giant cell tumor and Paget's disease of bone in one family: geographic clustering. Clin Orthop Relat Res.

[13] Kim GS, Kim SH, Cho JK (1997). Paget bone disease involving young adults in 3 generations of a Korean family. Medicine (Baltimore).

[14] Divisato G, Scotto di Carlo F, Pazzaglia L (2017). The distinct clinical features of giant cell tumor of bone in pagetic and non-pagetic patients are associated with genetic, biochemical and histological differences. Oncotarget.

[15] Hoch B, Inwards C, Sundaram M (2006). Multicentric giant cell tumor of bone. Clinicopathologic analysis of thirty cases. J Bone Joint Surg Am.

[16] Wang H, Wan N, Hu Y (2012). Giant cell tumour of bone: a new evaluating system is necessary. Int Orthop.

[17] Kattner KA, Stroink A, Gupta K (1998). Giant cell tumor of the sphenoid bone. Skull Base Surg.

[18] Huang L, Xu J, Wood DJ, Zheng MH (2000). Gene expression of osteoprotegerin ligand, osteoprotegerin, and receptor activator of NF-κB in giant cell tumor of bone. Am J Pathol.

[19] Yamamoto M, Fukushima T, Sakamoto S, Tomonaga M (1998). Giant cell tumor of the sphenoid bone: long-term follow-up of two cases after chemotherapy. Surg Neurol.

[20] Maloney WJ, Vaughan LM, Jones HH (1989). Benign metastasizing giant-cell tumor of bone: report of three cases and review of the literature. J Bone Joint Surg Am.

